# A Case of Dermatomyositis and Anti-EJ Autoantibody with Chronic Intestinal Pseudoobstruction Successfully Treated with Octreotide

**DOI:** 10.1155/2016/9510316

**Published:** 2016-11-03

**Authors:** Chiho Yamada, Shinji Sato, Noriko Sasaki, Takayoshi Kurabayashi, Sho Sasaki, Yasushi Koyama, Naofumi Chinen, Takayuki Wakabayashi, Yasuo Suzuki

**Affiliations:** ^1^Division of Rheumatology, Department of Internal Medicine, Tokai University, School of Medicine, Isehara 259-1193, Japan; ^2^Division of Rheumatology, Department of Internal Medicine, Tokai University Hachioji Hospital, Hachioji, Japan

## Abstract

Chronic intestinal pseudoobstruction (CIPO) is a serious complication in patients with connective tissue disease (CTD) and is sometimes life-threatening or fatal despite intensive medical treatment. Here, we report a patient with dermatomyositis (DM) and anti-EJ autoantibody who developed CIPO that was improved by octreotide. Because her abdominal pain and bloatedness were so severe and persistent, we introduced octreotide to relieve symptoms. In this case, continuous intravenous administration as well as long-acting subcutaneous injection of octreotide was effective for treating CIPO.

## 1. Introduction

Dermatomyositis (DM) is a systemic connective tissue disease characterized mainly by proximal muscle weakness and myalgia with typical skin rash (i.e., heliotrope rash or Gottron's papule) [1]. Chronic intestinal pseudoobstruction (CIPO) is a gastrointestinal disorder that resembles a mechanical obstruction of the bowels but without any physical blockage, and which is due to abnormalities of peristalsis [2]. Imaging reveals dilation of the small and large bowels with air-fluid levels. Connective tissue disease (CTD), particularly systemic sclerosis (SSc), is one of the underlying conditions associated with CIPO [3], whereas this syndrome is relatively rarely a complication of polymyositis (PM)/DM or systemic lupus erythematosus (SLE). To the best of our knowledge, for the first time, here we report a DM patient with anti-EJ autoantibody whose refractory CIPO was improved with octreotide. In this case, continuous intravenous administration with subsequent subcutaneous injection of octreotide was effective for amelioration of her abdominal symptoms.

## 2. Case Report

**Figure 1:**
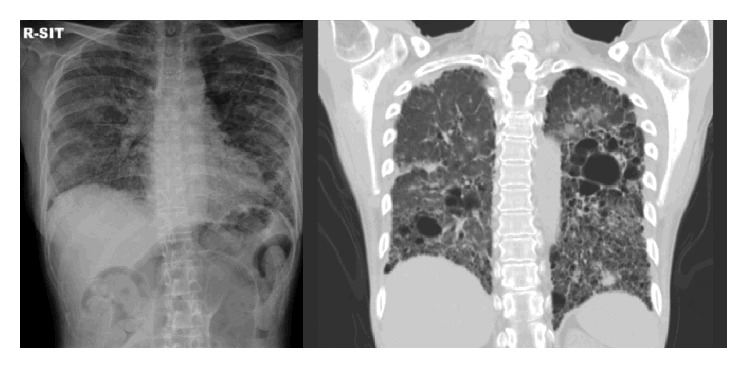
Chest radiography and CT findings at the emergent admission. Diffuse ground glass or reticular shadow with honey combing appearance in both lung fields.

**Figure 2:**
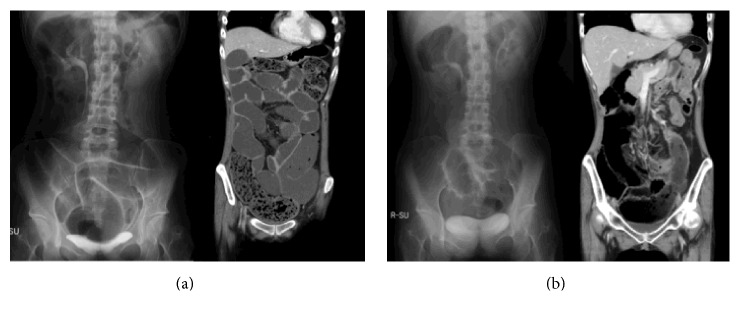
Abdominal radiography and CT findings at the emergent admission (a) and after treatment with octreotide (b). (a) Multiple and dilated small and large bowels with air-fluid levels in bowel loops (X-ray) and huge distention of bowel without mechanical obstruction (CT). (b) Thickening of the bowel wall and dilation of the bowels with air-fluid levels were improved.

A 38-year-old woman who was suspected of having interstitial lung disease (ILD) was referred to our hospital in July 2004. At that time, she had fever, heliotrope rash, and Gottron's papules and suffered from polyarthralgia and proximal muscle weakness. Laboratory examination revealed high serum creatine kinase (CK) levels and chest X-rays showed reticular and granular shadows on lower lung fields. On this basis, she was diagnosed as having DM with ILD and was hospitalized for initiation of treatment with 50 mg of prednisolone (PSL) daily. As her muscle symptoms and serum CK level improved, PSL was gradually tapered. In June 2006, when the PSL dose was down to 10 mg daily, her muscle weakness recurred. At the same time, she noticed a feeling of abdominal fullness and severe pain all over the abdomen; she was rehospitalized for further examination and treatment. Her abdomen was distended and tympanitic on percussion and bowel sound was absent on auscultation. Abdominal X-ray, CT, and endoscopy with a lower gastrointestinal tract fiberscope revealed an intestinal obstructive ileus with no discernible mechanical cause, leading to a diagnosis of CIPO. She was treated with conservative medical management using 20 mg of PSL, which gradually improved her symptoms. However, from this time on, she suffered sporadic persistent bloatedness and/or abdominal pain without the worsening of muscle or respiratory symptoms. In January 2012, she again suffered muscle weakness, dyspnea, and abdominal bloating and pain while taking 9 mg of PSL. She was urgently readmitted to our hospital. At admission, she had fever, proximal muscle weakness, myalgia, and dyspnea on exertion and arterial oxygen saturation decreased to 93% under oxygen inhalation (FiO_2_ was 0.35). Fine crackles were heard in both lower lung fields. The abdomen was distended and tenderness was present all over it. Bowel movement was hypoactive and a tympanitic percussion note was heard. Laboratory findings were white blood cell count 19,800/*μ*L, red blood cell count 546 × 10^6^/*μ*L, hemoglobin 12.3 g/dL, and platelets at 43.5 × 10^4^/*μ*L. Serum AST was 37 IU/L, ALT was 42 IU/L, LDH was 316 IU/L, and creatine kinase (CK) was 201 IU/L (normal range is up to 140 IU/L). Serum C-reactive protein and KL-6 were elevated to 0.49 mg/dL and 1383 U/mL, respectively. Anti-nuclear autoantibody was positive (×320, speckled pattern) as well as anti-U1RNP antibody and anti-SSA antibody. Immunoprecipitation assays revealed the presence of anti-EJ antibody. Chest X-rays and CT revealed diffuse ground glass or reticular shadows with honeycomb appearance in both lung fields, indicating the presence of ILD (Figure 1). Compared with previous CT imaging, however, obvious progression of ILD was not seen. Abdominal X-rays revealed multiple dilations of the small and large bowels, with air-fluid levels in bowel loops. Abdominal CT showed large distentions of the bowel in the absence of any mechanical obstruction (Figure 2(a)). As these findings strongly suggested relapse of myositis and CIPO, 20 mg of PSL, gastrointestinal prokinetic agents, and antibiotics were started. Fever, muscle symptoms, serum CK, and dyspnea and hypoxia improved relatively rapidly. However, despite treatment via inserted gastric tube to administer metoclopramide (30 mg/day), pantethine (90 mg/day), itopride hydrochloride (150 mg/day), mosapride acid hydrate (15 mg/day), daikenchuto (7.5 g/day), dimethicone (120 mg/day), lactulose (60 mL/day), magnesium oxide (2 g/day), and erythromycin (800 mg/day) for pseudoileus of CIPO, her bowel movement did not improve. In mid-March, we began continuous intravenous octreotide at 100 *μ*g for persistent CIPO. Soon after starting this treatment, peristaltic activity was detected, and subjective symptoms dramatically improved. Abdominal X-ray and CT also revealed that there was no thickening of the bowel wall and that dilation of the bowels with air-fluid levels was improved (Figure 2(b)). In the middle of April, although the route of octreotide administration was changed to subcutaneous (50 *μ*g for every 6 hours), she had no abdominal pain or distention and was discharged from hospital. The patient was treated with intramuscular long-acting octreotide (30 mg every 4 weeks) as an outpatient. She was well and was maintained in a stable condition with no abdominal symptoms on octreotide. In March 2013, octreotide was discontinued because she had been well for over 2 years. In September 2015, she died from an acute cerebral hemorrhage.

## 3. Discussion

To the best of our knowledge, this is the first case of a patient with DM and anti-EJ antibody developing CIPO and then being successfully treated with octreotide. The CTD most commonly associated with CIPO is systemic sclerosis; the case of other CTDs who develop CIPO over their clinical course seems relatively rare. Our patient manifested muscle weakness and typical DM rash but no sign of swollen hands or scleroderma and no history of previous Raynaud's phenomenon. Immunological examination revealed the presence of anti-EJ antibody, a myositis-specific antibody (anti-aminoacyl-transfer RNA synthetase (ARS) antibody). Taking these findings together, the patient was diagnosed as having DM. Although PM/DM patients sometimes suffer from dysphagia due to muscle weakness, dysfunction of visceral muscle is rarely seen. In our case, muscle symptoms improved relatively promptly, but abdominal disturbances were persistent after the improvement of muscle inflammation. However, CIPO can be a complication of PM/DM. Marie et al. reported a PM patient with CIPO complications, but without any sign of scleroderma [4]. The etiology of CIPO associated with PM/DM is still unknown and further identification of patients such as the case reported here would be required to determine this. As the cause of CIPO is various and our patient was complicated with ILD, one might suspect that CIPO arose due to chronic hypoxia. However, it was not likely that chronic hypoxia was cause of CIPO because her respiratory condition at the onset of CIPO was stable (blood oxygen saturation ranged from 96 to 98% in room air) and no progression of ILD was suspected.

We summarize available clinical and immunological characteristics of CTD associated with CIPO and treated with octreotide in Table 1 [3–16]. Of 24 CTD patients with CIPO, there were 20 (83%) with SSc or SSc/PM overlap syndrome, and of the remaining 4 two had SLE, one PM, and one DM (the latter, our case reported here). All patients received octreotide subcutaneously except for our case initially. This drug was effective for CIPO in 20 patients (83%). Nineteen (79%) had scleroderma, 15 (71%) experienced Raynaud's phenomenon, and 12 (67%) had ILD that might suggest the presence of fibrosis in the smooth muscle of the gastrointestinal tract. Twelve (50%) and 14 (79%) patients suffered from diarrhea and constipation, respectively. In our case, similar to most of the cases previously reported, gastrointestinal prokinetic agents or antibiotics did not improve abdominal manifestations. Therefore, we initiated octreotide intravenously, which resulted in improvement of CIPO. Although the administration route of octreotide in previous reports was subcutaneous or intramuscular [3–18], our case shows that continuous intravenous octreotide is also effective in CIPO associated with CTD. Moreover, experience with our case suggested that monthly subcutaneous administration of long-acting octreotide is also effective and has the advantage of ease of long-term management in the outpatient clinic. The efficacy and safety of octreotide in juvenile patients with CIPO was also reported [19]. The usefulness of antroduodenal manometry was suggested to evaluate octreotide response. Although we did not examine the manometry for the assessment of octreotide, the antroduodenal manometry would be a useful method to assess the effect of octreotide against CIPO.

In summary, here we report a DM patient with anti-EJ antibody who developed persistent CIPO. In this case, intravenous administration of octreotide relieved abdominal symptoms and subsequent application of long-acting octreotide achieved prolonged complete remission. Thus, this case emphasizes the importance of being aware of the possibility of CIPO in patients with DM, as well as documenting the efficacy of continuous intravenous octreotide.

## Figures and Tables

**Table 1:** Characteristics of CIPO complicated with connective tissue disease treated with octreotide.

	Diagnosis	Sex/age	Octreotide (dosage, routes of administration)	Effect	Scleroderma	Raynaud's phenomenon	Diminished esophageal peristalsis	ILD	Diarrhea	Constipation	Antibodies and other features
Soudah et al. 1991	SSc	M/63	50 *μ*g, SC	+	+	+	n.a.	+	+	+	Myopathy
	SSc	F/65	50 *μ*g, SC	+	+	−	n.a.	+	+	−	
	SSc	F/60	50 *μ*g, SC	+	+	+	n.a.	−	+	+	
	SSc	M/57	50 *μ*g, SC	+	+	−	n.a.	−	+	+	
	SSc	M/55	50 *μ*g, SC	+	+	+	n.a.	−	+	+	

Kobayashi et al. 1993	SSc	F/26	50 *μ*g, SC	+	+	+	n.a.	n.a.	+	−	

Lanting et al. 1993	SSc/PM	F/51	50 *μ*g, SC	+	+	n.a.	+	+	+	−	

Yamamoto et al. 1994	SSc	F/29	100 *μ*g, SC	+	+	n.a.	n.a.	+	+	+	*α*-RNP, myopathy

Ono et al. 1996	SSc	F/28	100 *μ*g, SC	+	+	+	n.a.	+	−	+	
	SSc	F/47	100 *μ*g, SC	+	+	+	n.a.	+	−	−	

Kanbe et al. 1996	SSc	F/61	100 *μ*g, SC	+	+	+	n.a.	+	−	+	

Ishikawa et al. 1999	SSc	F/66	SC^*∗*^	+	+	+	+	+	+	+	
	SSc/PM	F/35	SC^*∗*^	+^*∗∗*^	+	+	+	−	−	+	*α*-RNP, Ku

Perlemuter et al. 1999	SS	F/19	100 *μ*g, SC	+	−	−	n.a.	n.a.	−	+	*α*-RNP, SSA
	SLE	F/52	100–400 *μ*g, SC	+	−	−	n.a.	n.a.	+	+	*α*-RNP, DNA
	SSc	F/70	50–100 *μ*g, SC	+	+	+	+	n.a.	−	+	*α*-Scl-70, Jo-1, PM-1

Descamps et al. 1999	SSc/PM	F/53	75 *μ*g, SC	+	+	+	n.a.	n.a.	−	+	Dysphagia

Matsuki et al. 2000	SSc	M/64	50 *μ*g, SC	+	+	+	n.a.	+	−	+	
	SSc	F/65	100 *μ*g, SC	−	+	+	+	+	−	+	*α*-Scl-70

Marie et al. 2001	PM	M/55	50 *μ*g, SC	+	−	−	−	−	−	+	

Malcolm and Ellard 2001	SSc	F/75	50 *μ*g, SC	−	+	+	−	+	+	−	Intestinal perforation

Suzuki et al. 2005	SSc/PM	F/31	100 *μ*g, SC	−	+	+	+	−	−	+	*α*-Ku

Leonardi et al. 2010	SLE	F/51	50 *μ*g, SC	−	−	n.a.	+	n.a.	−	+	PSL pulse was effective

Our case 2016	DM	F/38	100 *μ*g IV, 50 *μ*g SC, 30 mg IM	+	−	−	+	+	+	+	*α*-RNP, SSA, EJ

^*∗*^Dosage was not available, ^*∗∗*^partial response.

ILD: interstitial lung disease; SSc: systemic sclerosis; SS: Sjögren's syndrome; PM: polymyositis; DM: dermatomyositis; SLE: systemic lupus erythematosus; n.a.: not available.
